# Microfabrication and Integration of a Sol-Gel PZT Folded Spring Energy Harvester

**DOI:** 10.3390/s150612218

**Published:** 2015-05-26

**Authors:** Jonathan Lueke, Ahmed Badr, Edmond Lou, Walied A. Moussa

**Affiliations:** 1 Department of Mechanical Engineering, University of Alberta, University of Alberta, Edmonton, AB T6G 2G8, Canada; E-Mail: lueke@ualberta.ca; 2 Department of Electrical and Computer, University of Alberta, University of Alberta, Edmonton, AB T6G 2V4, Canada; E-Mails: abadr@ualberta.ca (A.B); elou@ualberta.ca (E.L.)

**Keywords:** PZT sol-gel, piezoelectric energy harvesting, conditioning circuitry, microfabrication, MEMS, packaging, integration, feasibility study

## Abstract

This paper presents the methodology and challenges experienced in the microfabrication, packaging, and integration of a fixed-fixed folded spring piezoelectric energy harvester. A variety of challenges were overcome in the fabrication of the energy harvesters, such as the diagnosis and rectification of sol-gel PZT film quality and adhesion issues. A packaging and integration methodology was developed to allow for the characterizing the harvesters under a base vibration. The conditioning circuitry developed allowed for a complete energy harvesting system, consisting a harvester, a voltage doubler, a voltage regulator and a NiMH battery. A feasibility study was undertaken with the designed conditioning circuitry to determine the effect of the input parameters on the overall performance of the circuit. It was found that the maximum efficiency does not correlate to the maximum charging current supplied to the battery. The efficiency and charging current must be balanced to achieve a high output and a reasonable output current. The development of the complete energy harvesting system allows for the direct integration of the energy harvesting technology into existing power management schemes for wireless sensing.

## 1. Introduction

The focus of the majority of Microelectromechanical (MEMS)-based energy harvesting research is the optimization of the power output of a specific energy harvester. Regardless of the harvesting methodology or design [1,2,3,4], significant challenges exist in the design, fabrication, and implementation of the energy harvester. This paper will focus on the challenges encountered in the microfabrication; packaging and integration of a fixed-fixed folded spring vibration-based piezoelectric energy harvesting system [5]. The focus of this specific design of the folded spring energy harvester was to controllably and reliably reduce the natural/operational frequency of the harvester while remaining insensitive to out of plane static deformations due to residual stresses [5,6,7,8,9]. The folded spring allows for the effective length of the spring to be increased while allowing for free expansion, contraction, and rotation of individual beam elements of the spring to dissipate residual stresses.

In general, there are two methodologies available to fabricate the released structures required for the piezoelectric energy harvesters. The first method relies upon surface micromachining techniques to undercut the released structures [4,10,11,12,13,14]. Typically, this method uses a sacrificial material and an etch stop, such as a Silicon-On-Insulator (SOI) wafer, to release the structural members of the device. This fabrication methodology is capable of producing thin structural beams with high accuracy, which is desirable for low frequency energy harvesting applications. The second method relies on bulk micromachining to define the geometry and release the structure of the harvesters [4,15,16,17,18]. Typically, this method uses Deep Reactive Ion Etching (DRIE) to remove the structural silicon from both sides of the wafer to define the structural beams and proof masses required by the energy harvesters. Although the bulk micromachining methodology may introduce difficulties in fabricating thin structural members, integrated proof masses are difficult to achieve with surface micromachining techniques. A release methodology was developed for the energy harvesters designed in this research [5,9], based upon the bulk micromachining techniques, to allow for the required large proof masses and thin structural beams. Several difficulties encountered with this chosen method of releasing the energy harvester structure will be discussed at length in the following sections. Throughout the remaining microfabrication process flow, several obstacles were encountered, including difficulties caused by titanium residues from lower electrode etching, uneven and non-continuous films deposited by a simultaneous sol-gel and lift-off process, and wafer handling issues caused through the backside etching required to release the harvesters from the silicon wafer [9,19].

Additionally, the harvester operates under a base vibration, wafer-level testing was not possible. Therefore, full packaging of the harvesters were required, leading to several challenges dealing with adhesion of wirebonds and the overall design and implementation of suitable packaging [9,19]. Finally, significant challenges were encountered in designing and fabricating suitable conditioning circuitry including maximizing the efficiency and charging current delivered to the storage medium. Additionally, the relationship between the efficiency and battery charging current of the conditioning circuitry in response to variations in the input voltage, input frequency, and battery voltage level will be examined with a feasibility study. In the following sections, each of the microfabrication, packaging and integration and conditioning circuitry-based challenges will be examined in detail.

## 2. Microfabrication of PZT Sol-Gel Folded Spring Energy Harvesters

### 2.1. Energy Harvester Microfabrication Process Flow Overview

**Figure 1:**
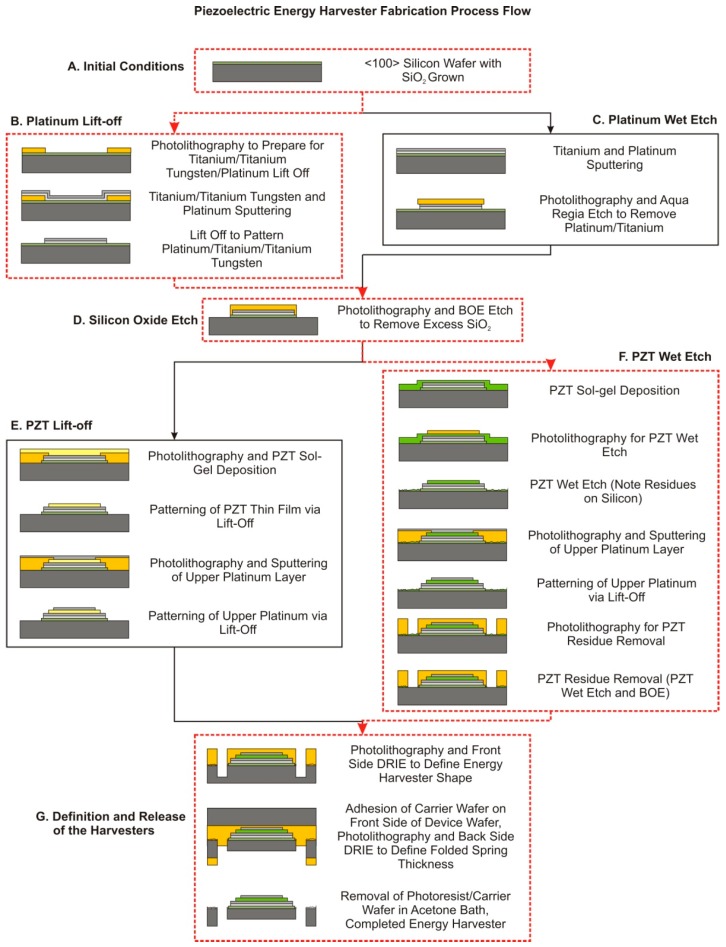
The PZT sol-gel folded spring energy harvester microfabrication process flow. The optimum arrangement of fabrication processes is highlighted in red [9].

The process flow developed in [5,9] to microfabricate PZT sol-gel folded spring energy harvesters has undergone several iterations of development in order to overcome a variety of fabrication-based challenges. In response to these fabrication obstacles alternate fabrication processes were developed for multiple fabrication steps, including the lower electrode deposition, PZT sol-gel deposition, PZT patterning, and the definition and release of the energy harvesters. The entire fabrication flow is shown, with the optimum arrangement of the fabrication flow highlighted, in Figure 1 [9].

The fabrication flow shown in Figure 1 is capable of producing piezoelectric energy harvesters of the same general cross section, of any configuration and beam thickness, using any sol-gel based piezoelectric film with suitable adhesion layers. The microfabrication fabrication process begins with a double side polished prime <100> silicon wafer. The thickness and uniformity of wafer chosen for this process will have a significant effect on the natural frequency of the harvesters, the volume of proof masses, and variation in folded spring thickness. Therefore, it is important to have wafers with as uniform thickness as possible. A layer of silicon oxide was then deposited with dry oxidation, as an electrical isolation barrier, diffusion barrier, and adhesion layer for the subsequent thin films. The deposition of lower electrodes can occur in two methods. The first method, as shown in “B. Platinum Liftoff” in Figure 1, uses a negative photoresist as a sacrificial material to lift off the required lower electrode layers. This option allows for the use of any lower electrode material, assuming adhesion to the underlying silicon oxide layer. The second procedure, as shown in “C. Platinum Wet Etch”, uses an Aqua Regia Etch to pattern the titanium and platinum layers. Regardless of procedure used to pattern the lower electrodes of the piezoelectric stack, a buffered oxide etch is required to pattern the exposed silicon oxide layer. There are two methods available to deposit the required sol-gel PZT layer and the upper electrodes of the piezoelectric stack. The first, as shown in “E. Lift-off” in Figure 1, involves the sequential lift off of the PZT material and upper platinum electrode. The second, as shown in “F. PZT Wet Etch” in Figure 1, involves the sol-gel deposition and wet etch of the PZT and subsequent lift off of the upper platinum electrode. The microfabrication of the energy harvester is completed with a combination of two DRIE. The first etch, from the front of the wafer, defines the planar geometry of the energy harvester. The second etch, from the backside of the wafer, releases the structure of the energy harvesters and defines the overall thickness of the folded springs. The second DRIE etch also allows for the use of cleaving trenches to separate individual released energy harvesters from the wafer without the use of potentially harmful dicing.

Three major challenges were encountered in the development of this microfabrication process—overetching of the titanium films leading to the modification of the lower electrode deposition process from etching to lift off; the poor coverage, limited thickness and peeling of the PZT sol-gel film leading to an alternate PZT material and wet PZT etch; and the release and conservation of the rectangular cross section of the folded spring leading to a two-step DRIE release procedure. In each case, the resolution to the encountered obstacle resulted in a modification to the process flow in Figure 1.

### 2.2. Lower Electrode Deposition Process Challenges

**Figure 2:**
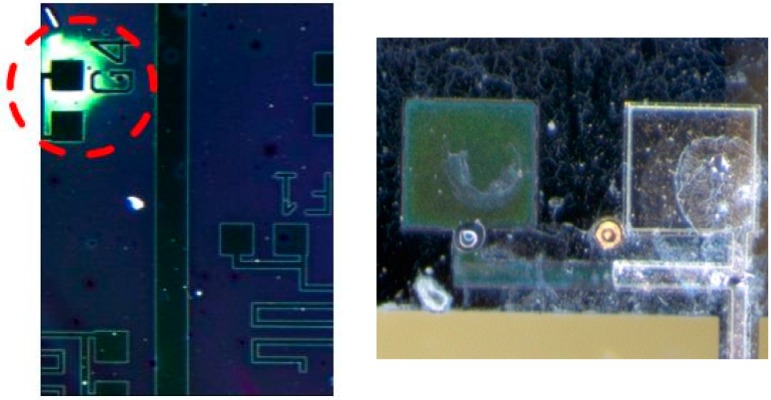
Platinum and titanium films etched by aqua regia. Residual titanium film from underetching is highlighted in the left figure, causing the overetch seen in the right figure in the following buffered oxide etch [9].

The first microfabrication-based challenge encountered involved the lift off of titanium films due to uneven etching and deposition of the platinum and titanium films pre-deposited on the wafer. The initial iteration of the microfabrication process included an aqua regia wet etch to take advantage of the high etch rate of the etch, upwards of 13 nm/min. Typically, the isotropy of the aqua regia wet etch is not a drawback, however, when coupled with the non-uniformity of titanium/platinum sputtering, on the order of a few tens of nanometers across the wafer, the situation seen in Figure 2 can arise.

As highlighted in Figure 2, the nonuniformity in deposition across the wafer will later manifest as both underetched and overetched films. In areas of locally thicker titanium films, residual films will occur while the remainder of the wafer is properly etched. It is possible to remove the residual titanium, however, the correctly etched features will be overetched and removed. Alternatively, if the residual titanium film is not removed, the lower electrodes would be irreversibly damaged.

The etch rate of titanium in Buffered Oxide Etch (BOE) is extremely high [20], therefore any exposure to the BOE etchant in any subsequent step would cause the lower electrodes of the harvester to lift off from the wafer, as shown in Figure 2.

**Figure 3:**
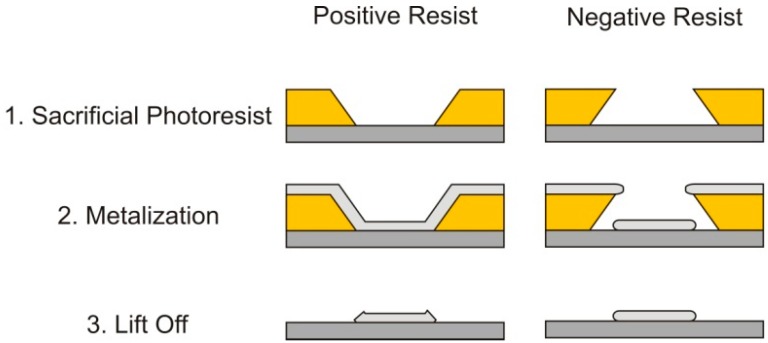
A schematic outlining the stages of the lift off process with both positive and negative photoresists. Sidewall angles are exaggerated for illustration [9].

To rectify this issue, a lift off procedure was developed, using a negative photoresist as a sacrificial layer. The goal of this sacrificial layer is to prevent the adhesion of the sputtered metal to the wafer. After the metal is deposited, the photoresist is dissolved, and the unwanted material is removed from the wafer, as shown in Figure 3.

For the lift off application, the polarity of the resist is not specifically important—the cross sectional profile of the resultant photoresist pattern is important. When a positive photoresist is used for lift-off, the cross sectional profile that is achieved is shown above in Figure 3. Although the sidewall angle is not as exaggerated as shown in Figure 3, the sidewalls will be coated with the sputtered metal, creating a continuous metal film. In order to complete the lift off procedure with a positive photoresist, ultrasonic cleaning and other harsh means are required to remove the unwanted metal. Alternatively, the sidewall profile of a negative photoresist aids in the liftoff patterning of the metallic film, preventing the metal layer from being deposited on the sidewalls of the photoresist. This allows for the lift off to occur easily and much more accurately. Additionally, the use of a robust lift off procedure allows for a variety of adhesion materials to be used to both overcome additional fabrication issues encountered later in the fabrication process and interchange piezoelectric materials in this process flow if required.

### 2.3. PZT Sol-Gel Deposition and Patterning Challenges

The second microfabrication challenge encountered in the fabrication of the harvesters was the poor adhesion and coverage of the PZT film. The initial development of the harvester, as shown as Section “E. PZT lift-off” in Figure 1, utilized a lift-off to pattern the sol-gel PZT film. This method worked well to develop the understanding of the sol-gel deposition procedure; however, there were significant film quality issues encountered. Due to the anneal/bake requirements of the sol-gel PZT and the thermal budget of the sacrificial photoresist, the lift-off and deposition must occur simultaneously. A sacrificial layer of photoresist is deposited and patterned to prevent the PZT film from adhering to the wafer in areas that the PZT film is not required. After the photoresist is patterned, the PZT sol-gel was then spun on to the wafer. Approximately 5 mL of the PZT sol-gel precursor is spread on the wafer at 250 RPM for 20 s, and then spun at 1000 RPM for 30 s. Once the spinning of the PZT precursor is complete, the wafer must be baked at 100 °C for 15 min to evaporate the sol-gel precursor solvent. When the baking is complete, a partial anneal is required in order to solidify the film sufficiently to allow for lift off to occur. The anneal is ramped to 150 °C and held for 15 min. After the partial anneal was complete, the temperature was ramped down to ambient. The wafer was immersed in acetone to remove the sacrificial photoresist and perform the PZT lift off. The PZT sol-gel film is partially soluble in acetone; therefore, it was necessary to limit the total exposure to limit the volumetric loss of the film. The optimum lift off occurred with a 5 min soak in acetone, followed by a 2 min, mid power, ultrasonic bath. After the lift off process was complete, the wafer was annealed to crystallize the PZT film. To prevent cracking from thermal shock, the crystallization anneal was also ramped to 475 °C, as required for this specific sol-gel material. The PZT film was annealed for one hour, and then ramped down to ambient over several hours. If the annealing furnace was opened prematurely, the thermal shock of the ambient air rushing into the furnace would damage the PZT film. This method of PZT deposition was capable of producing films as thick as 3 µm with repeated depositions.

**Figure 4:**
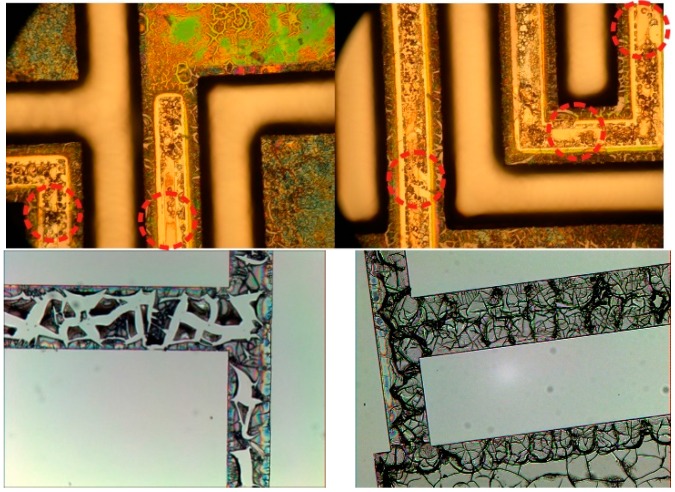
Examples of poor quality PZT film patterned by the lift off procedure. Circled areas denote short circuits and voided film.

During the lift-off, it was found that the semi-solid PZT film would locally dissolve from the wafer when exposed to acetone. This caused a net volume loss, which ultimately led to a contraction of the PZT film during annealing, causing the voids and cracks that are seen in Figure 4.

To fill these voids shown in Figure 4, additional layers of PZT are required. However, to add additional layers of PZT, the entire lift-off process would be repeated. Although a fairy thick film is possible with this initial PZT sol-gel material and deposition procedure, the surface roughness and unevenness of the film makes deposition and continuity of the upper electrodes difficult. When it was apparent that an alternative PZT sol-gel precursor could allow for better coverage and uniformity, a second deposition/patterning technique was developed, as shown in “F. PZT Wet Etch” in Figure 1. The second PZT material, from Mitsubishi Materials Corporation (Schaumburg, IL, USA), required a much higher crystallization temperature of 700 °C for crystallization. Therefore, the lift-off process that was developed in the previous section would not be suitable for patterning. To pattern the second PZT material, a wet etch was selected from literature and then adapted for use in our fabrication facility [9,21].

**Figure 5:**
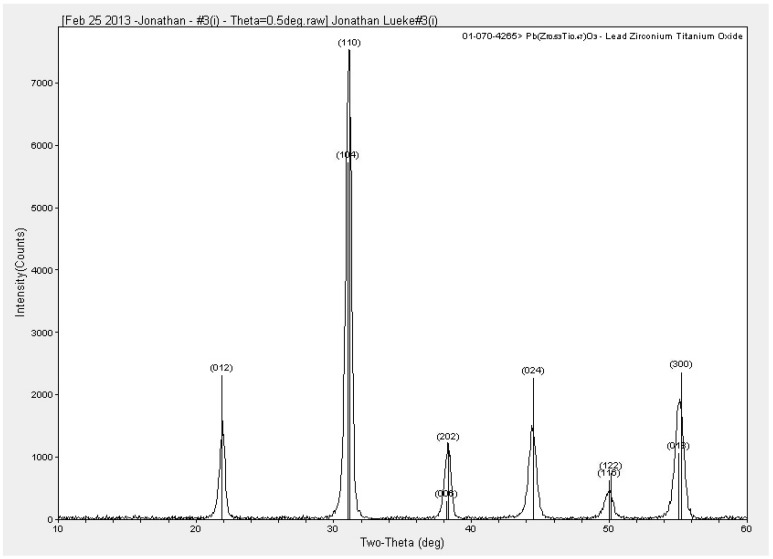
X-Ray Diffraction of the Mitsubishi Materials PZT material. This XRD is for a sample of 0.24 μm [9].

The second PZT sol-gel deposition procedure was similar to the first deposition procedure, with some minor material dependent alterations. In this case, a repeated spin-bake process was required to build up the thickness of the film. The PZT sol-gel was spun on the wafer at 500 RPM for 5 s and then spread on the wafer at 3000 RPM for 30 s. Once the PZT sol-gel was spun, the film was baked on a hot plate at approximately 350 °C to solidify the film. At this point, this spin-bake process must be repeated three times to build up 0.24 μm of PZT, in steps of 0.08 μm. Once the spin-bake cycle was completed, the film was annealed to crystallize the PZT. The anneal takes place at 700 °C for 15 min. Once annealing is complete, the entire deposition process must be repeated to build up the film thickness in steps of 0.24 μm. In this work, PZT thicknesses of up to 0.96 μm have been achieved. Through X-Ray diffraction (XRD), the proper crystal structure of the PZT film has been confirmed, as shown in Figure 5.

As shown in Figure 5, the expected peak locations for the crystal planes of the PZT film are shown as labelled vertical lines. The peaks of the XRD measurement correspond with the expected crystal planes, with the maximum peak aligned with the (110) peak of the PZT standard, suggesting that the deposited PZT film is aligned to the (110) crystal plane.

**Figure 6:**
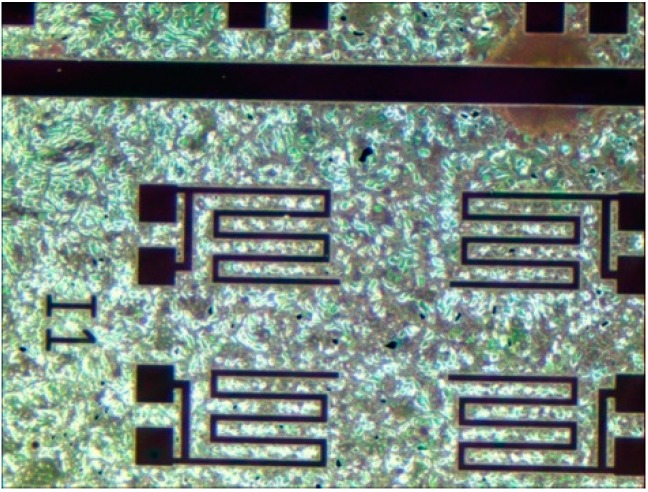
Correctly formed PZT thin film, continuous on the exposed platinum, cracked/discontinuous on the exposed silicon/silicon oxide [9].

On areas of exposed silicon and silicon oxide, the PZT film tended to crack and produce discontinuous films. Ultimately, this is more of a cosmetic problem and does not cause any performance-related issues. An example of an annealed PZT film using this process can be seen in Figure 6.

As shown in Figure 6, the PZT sol-gel film forms correctly and continuously on the adhesion layers of platinum and titanium tungsten. On materials that the PZT is not expected to adhere well to, such as exposed silicon and silicon oxide, the film is rough and shows small cracks and discontinuities. This behavior has no impact on the energy harvesters, since the film in question is far from the physical locations of the harvesters and will be removed in a subsequent wet etch step.

An additional PZT-based challenge was encountered through the adaption of the PZT sol-gel deposition process. The crystallization anneal for this PZT film occurs at the same temperature titanium based silicides begin to form, ~700 °C [22]. Therefore, as the wafer is annealed, the titanium diffuses from the titanium layer into the silicon oxide, reducing the thickness and degrading the adhesion of the titanium layer. Given enough time in the annealing furnace, the titanium layer is sufficiently removed to cause the PZT to peel off the wafer due to residual stresses and poor adhesion between the remaining platinum and silicon oxide. To overcome this adhesion problem, the titanium layer was replaced with a titanium tungsten. The larger tungsten atoms act as a diffusion barrier, not allowing the titanium to freely migrate out of the film during the annealing processes. This prevents most of the titanium loss, maintaining the thickness and adhesion of the films, allowing for the deposition of continuous PZT films without major cracks/voids through multiple applications of the PZT sol-gel.

**Figure 7:**
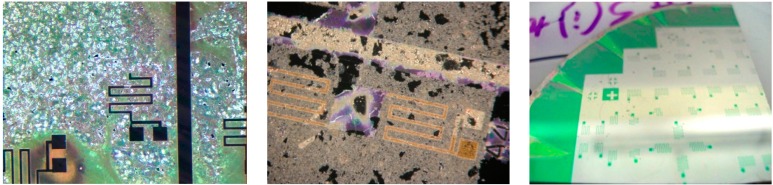
The PZT film during the etch process. The left figure shows the unetched PZT film. The middle figure shows the intermediate residue left after the second etch process. The right one shows the completed etch. PZT deposited on platinum appears dark green [9].

As was discussed previously, lift-off of this specific PZT material was not possible. Additionally, due to cross contamination concerns, dry plasma etching of PZT was not available. Therefore, a wet etch technique was chosen from literature, and adapted for use in our fabrication facility [21]. The wet etch adapted from literature is composed of three separate wet etches [21], the intermediate stages and final etch result are shown in Figure 7.

The initial unetched state of the unetched PZT film is shown in the left of Figure 7. First, the PZT is etched in a solution of Buffered Oxide Etch, hydrochloric acid, ammonium chloride, and water (1BOE:2HCl:4NH_4_Cl:4H_2_O) to convert the open PZT material to a white residue of PbClF [21]. In comparison to other available PZT wet etch procedures, the addition of the salt ammonium chloride (NH_4_Cl) helps reduce the problematic undercut caused by the BOE etch, slowing the etch rate slightly, minimizing the lateral etch [21]. Second, the PbClF residue is etched with a solution of nitric acid and water (2HNO_3_:1H_2_O) producing a residue of water soluble PbCl_2_ [21]. The middle of Figure 7 shows the remaining water soluble PbCl_2_. Lastly, a prolonged soak in deionized water is used to remove the water soluble PbCl_2_, resulting in the etched film shown in the right of Figure 7. This etch process is specifically designed to etch the PZT that is present on the platinum electrodes only—not the film deposited on exposed silicon and silicon oxide. Therefore, to prevent cross contamination of lead in equipment used in subsequent plasma etches, an additional PZT wet etched followed by an additional buffered oxide etch is required to remove the residual PZT film.

**Figure 8:**
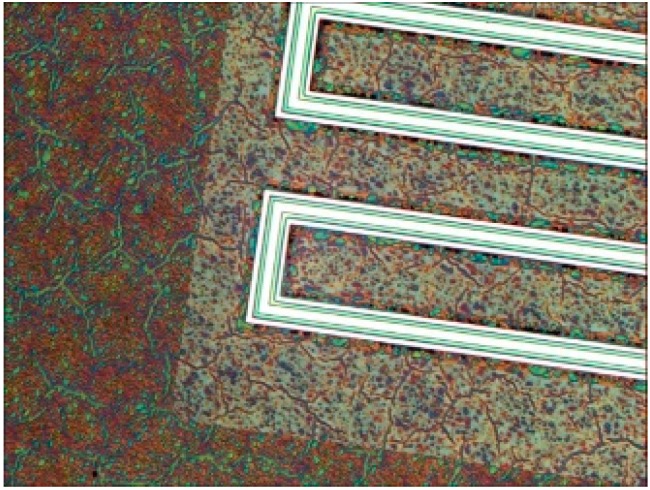
Mid-process photos of the second PZT etch needed to remove the residual PZT film from the silicon/silicon oxide. A color change in the PZT residual film denotes a height change from etching [9].

As shown in Figure 8, the etch area for this process is far from the piezoelectric stack which is distinguishable by the continuous platinum and PZT layers.

### 2.4. Challenges Involved with Definition and Release of the Energy Harvesters

**Figure 9:**
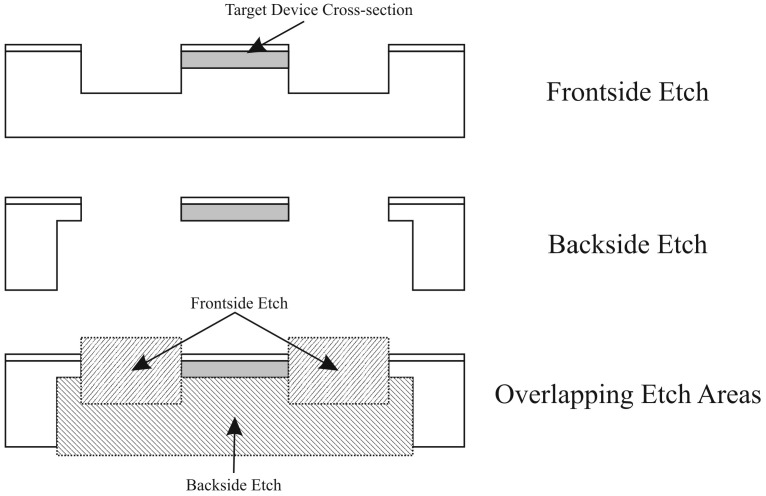
Overlapping Etch Areas releasing and defining the cross section of the harvesters [9,19].

The last microfabrication challenge encountered in the development of the harvesters involved the release of the harvesters and preservation of the cross section of the structural members of the harvesters. As shown as Section “G. Definition and Release of the Harvesters” in Figure 1, two sequential deep reactive ion etches (DRIE) were required to define the geometry of the folded springs and release the harvesters. The process uniformity of the DRIE process coupled with the thickness variation of the silicon wafer selected for fabrication can cause a significant difficulty in uniformity and accuracy of the etch depth. The DRIE non-uniformity is a function of the overall depth, therefore the total error in uniformity and etch depth increases cumulatively with etch depth [23,24,25,26,27]. This variability ultimately causes individual devices, of the same design on the same wafer, to have differing natural frequencies as discussed in [5]. There have been some attempts to tune the parameters of the DRIE process, such as RF power, chemistries used in the etch, cooling backpressure, dummy structures, to achieve a more consistent and accurate etch [23,24,25,26,27,28]. The methodology used to define the planar geometry and release the energy harvesters is based upon overlapping the etched volumes created from two sequential etch steps, as shown in Figure 9.

The two-step methodology, shown in Figure 9, begins with a deep front side DRIE (at least 100 µm) to define the planar geometry, followed by a deep backside DRIE to define the thickness of the folded springs. The depth of the front side etch is significantly larger than the desired thickness of the energy harvester to ensure that the folded springs of the harvester have near vertical sidewalls. The deep backside DRIE then completes the near-rectangular cross section of the folded silicon beam. As shown in [5], the undercut of this etch can significantly alter the stiffness of the beam, deviating the natural frequency of the system from the expected design. The methodology used to release the energy harvesters through the combination of etch steps adds a major wafer handling difficulty. Since deep wells are etched through the wafer along crystal planes in the final etch process, the device wafer becomes progressively more delicate. Typically, the DRIE process requires the wafer to be gas cooled to maintain a constant etch rate. The slight pressure differential between the near-vacuum required for the plasma and the few millitorr of helium required to cool the wafer will cause the increasingly delicate wafer to burst during the final stages of the etch process. If the wafer survives the final DRIE etch, the weakened wafer can spontaneously cleave along etched areas and/or major crystallographic planes. Due to this problem, it was necessary to stabilize the device wafer with a carrier wafer previous to the final etch step.

To prevent damage and autocleaving during the final DRIE release etch, a carrier wafer was adhered to the devices wafer using photoresist as an adhesive. Although there are multiple methods to adhere the device and carrier wafer, including photoresist, Crystalbond adhesive, and double sided thermal release tape, it was found that photoresist was the only method available capable of sufficient heat transfer to cool the etched surface of the device wafer [9,29]. As the wafer is etched, both the mechanical etching and the plasma heat the top surface of the wafer. Silicon, having a high thermal conductivity, transfers the heat almost instantaneously. In typical situations, the backcooling of the DRIE process is tuned to offset the heating of the etch such that the photoresist mask does not exceed its maximum allowable temperature. If the resist is heated above its maximum temperature, the resist will degrade, either reflowing, losing the pattern of the mask completely, or irreversibly adhering to the surface of the wafer. This typically causes poor quality etching, non-uniformities, etch artifacts, global wafer etching, and limited etch depth. The photoresist-based method was the only one to perform suitably in both adhesion and heat transfer, therefore, it will be used as the main method of adhering the carrier wafer.

**Figure 10:**
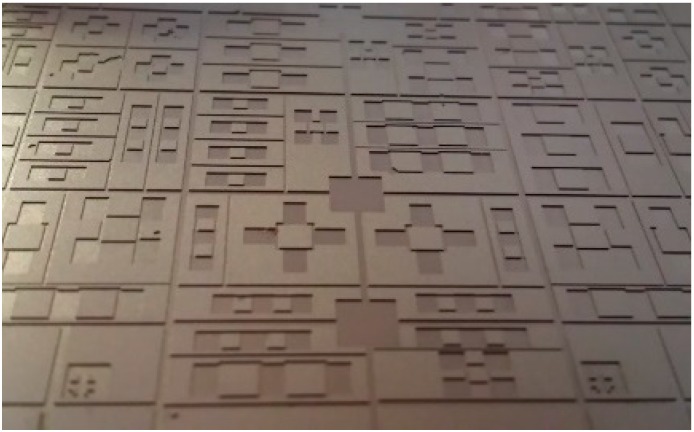
Cleaving trenches and backside wells etched on the backside of a testing wafer [9].

The use of a carrier wafer allows for—the separation of individual harvesters for packaging and testing without damaging the harvesters. The most common method of separating MEMS devices, dicing, is not well suited for this application. Dicing the wafer could adversely affect the overall yield of the harvesters by damaging the flexible, released structures. In the dicing process, a high speed rotary diamond blade, lubricated by a water jet, is used to cut the wafer into smaller sections. Both the vibration caused by the cut and the force of the water jet can cause serious damage to the individual energy harvesters during dicing. Cleaving can be used as an alternative for dicing to separate individual energy harvesters [19,30,31,32,33]. For this specific application, the use of a carrier wafer in the final DRIE etch step in the microfabrication process shown in Figure 1, allowed for the addition of deep cleaving trenches to the final backside etch pattern. The trenches integrated into the backside well pattern, as shown in Figure 10, followed major crystallographic planes of the silicon and were etched to the same thickness as the folded springs.

To remove the carrier wafer, both wafers are immersed in acetone and soaked for at least 24 h. The immersion in acetone completely dissolves all photoresist present. Additionally, the low surface tension of the acetone prevents any mechanical damage due to stiction during the separation of the wafers. Once the wafers are separated, the device wafer must be removed from the acetone bath and placed in a deionized water bath. The bottom of the bath should be lined with a soft material, such as cleanroom wipes, in order to provide a semi-flexible surface for cleaving. The cleaving must take place under water so that the energy released by the cleaving is damped sufficiently to prevent device damage. Once the wafer is submerged, the wafer is cleaved into sequentially smaller pieces until each harvester is separated. Light pressure applied by a sharp object will provide sufficient force to cleave the wafer along the etched cleaving trenches. This cleaving procedure was the precursor to the work published in [33].

## 3. Packaging and Integration of the Energy Harvesters

Although are several packaging schemes available for MEMS-based devices, there are limited complete packaging solutions for vibration-based energy harvesting. The energy harvesters operate under a base excitation; therefore it was not possible to directly connect to the energy harvesters in a typical probing station. The packaging scheme must allow for unhindered movement of the harvester to prevent damage and electrical losses. Additionally, suitable electrical connections must be made to the harvester to capture the voltage created through the vibration of the structure. Finally, the packaged energy harvester must be integrated into conditioning circuitry capable of efficiently rectifying and storing the harvested power.

### 3.1. Overview of the Packaging Methodology for the Energy Harvesters

**Figure 11:**
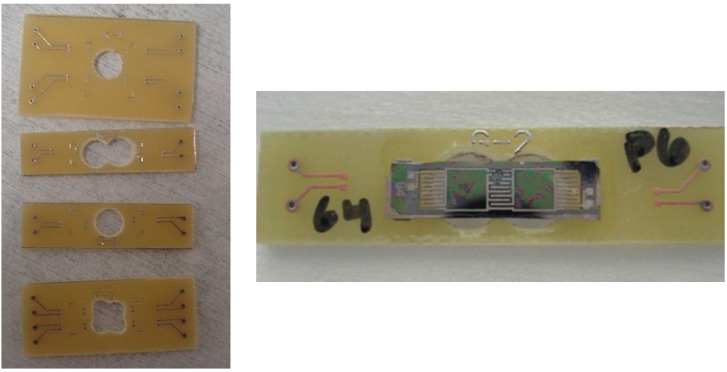
Solid PCBs developed for harvester packaging. (**Left**) separated and prepared individual PCBs; (**Right**) a mechanically packaged harvester on an individual PCB [9].

It was chosen to package the harvester onto printed circuit boards to enable *in-situ* characterization. Cyanoacrylate glue is used to mechanically adhere the piezoelectric energy harvester onto the printed circuit board (PCB). The cyanoacrylate glue is biocompatible, and strongly adheres the silicon base of the energy harvester to the PCB. As shown in Figure 11, in order to allow free vibration, a hole is punched in the PCB where the proof mass of the harvester is expected to be located. This ensures that the energy harvester will not contact the circuit board during operation, preventing harvesting losses and potential damage.

**Figure 12:**
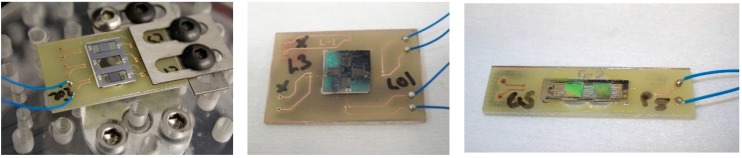
Several examples of fully packaged Class II Harvesters ready for testing [9].

The electrical connections between the energy harvester and the outside world are made with ball-wedge wirebonds and soldered wires. The wirebonds are used to create the intermediate electrical connection between the energy harvester and the PCB. The solder paste layer on the PCB is removed in order to wirebond directly to the copper trace. When material compatibility, bond adhesion and bond direction are a challenge, ball-wedge bonding is a more suitable choice. For example, for energy harvesters using platinum for electrode layers, the ball-wedge bond is more suitable to overcome the difficult adhesion to platinum. Additionally, ball-wedge bonding provides additional flexibility by allowing bonding at low ultrasonic bonding power, overcoming material compatibility issues such as fragile/rough structures, including the platinum/PZT/platinum layers produced in the research. The use of a ball bond allows for the decrease of applied ultrasonic energy to the platinum/PZT/platinum stack, preventing the possible delamination of electrodes. Generally, achieving good bond quality and adhesion is the major difficulty in wire bonding of energy harvesters. This can be significantly improved by tuning the bonding parameters to suit the electrode materials. These parameters include the bonding ultrasonic power, ultrasonic time, diameter of ball bond, and applied force [34]. Tuning these parameters for specific pad materials and conditions allows for the reliable and consistent bonding. With ball-wedge wirebonding, typically, the ball, or start of the bond is reserved for the pad that is more difficult to bond to. In this case, it was advantageous to use the wedge-end of the bond on the platinum/PZT/platinum stack. Heating applied to the bonding capillary was increased, in order to make the gold wire more malleable during bonding allowing for a significant decrease in applied ultrasonic energy required for the bond. This allowed for the wedge bond on the top platinum electrode to be completed with minimum ultrasonic energy applied, preventing delamination of the upper electrode. The connections to the outside world were provided by wires soldered to the PCB. These electrical connections do not to add a parasitic capacitance that would reduce electrical output. Examples of packaged harvesters can be seen in Figure 12.

**Figure 13:**
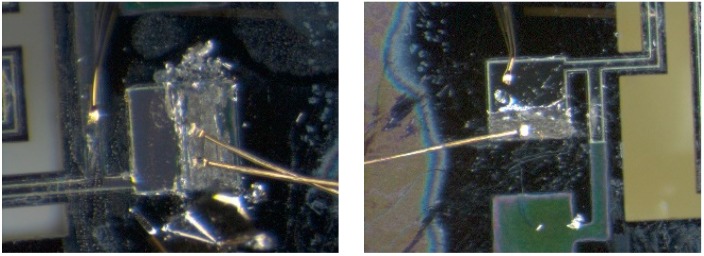
Examples of removing platinum and PZT from the remaining electrode of a damaged harvester to allow for packaging [9].

The wirebonding scheme developed in this work also allowed for the reclamation of the harvesters that were damaged through overetching encountered in the development of the microfabrication process. The overetch completely removed the exposed platinum-only electrodes of the harvesters leaving only the platinum/PZT/platinum stack electrode. To gain access to both electrodes, the upper platinum electrode and PZT material was carefully removed by hand. This allowed for access to both upper and lower electrodes for wirebonding, as shown in Figure 13. The remaining electrode pad area is large enough to allow for bonding to both upper and lower electrodes. The wirebonding procedures developed were not altered to fully package these salvaged harvesters. The ability to salvage damaged harvesters further demonstrates the robustness of this packaging scheme.

### 3.2. Overview of the Conditioning Circuitry for the Energy Harvesters

**Figure 14:**
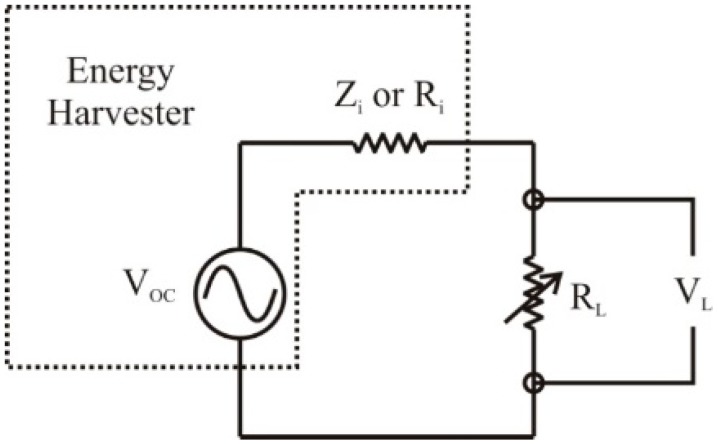
The simple circuit consisting of the energy harvester and a variable resistor used for initial characterization [9].

Conditioning circuitry is required to complete the integration of the energy harvester for characterization, testing, and later use in power management systems. In order to measure the output power of the harvester and determine the optimum load resistance, a simple circuit consisting of the harvester and a variable resistor, shown in Figure 14, was required.

For maximum power transfer, the internal, or input resistance of the voltage source must match the load resistance [35]. As can be seen in Figure 14 the energy harvester can be idealized as an AC voltage source and a resistor symbolizing the real component of the internal impedance of the harvester (Z_i_ or R_i_). The internal impedance of the harvester is a function of the operational frequency of the harvester and the capacitance formed across the deposited PZT material. When the load resistance matches the input resistance of the harvester, the harvester will transfer maximum output power to the circuit. The output voltage of the harvester can be measured across the load resistance (R_L_) to calculate the total output power of the harvester. This circuit was used to confirm the operation of the harvesters and to determine the maximum output power available from the harvester.

**Figure 15:**
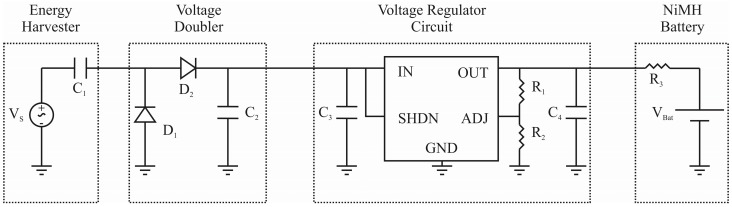
The full energy harvesting system developed in this study allowing for the storage of the power produced by the energy harvesters.

For more complex characterization and integration into power management systems, a second conditioning circuit is required in order to convert the harvested AC signal into a storable DC voltage. Several conditioning circuits are reported in literature, divided into passive [36,37,38] and active [39,40,41,42] conditioning circuits. Passive conditioning circuitry uses a simple configuration of a small number of passive components for the AC/DC rectification. In contrast, active conditioning circuits require active components, such as Metal Oxide Semiconductor Field Effect Transistor (MOSFETs), to rectify the AC signal. Although the conversion efficiency of active conditioning circuitry is higher, additional components such as a feedback circuit, are required. In this feasibility study, a passive conditioning circuit was used. Figure 15 shows a complete energy harvesting system which consists of the energy harvester, a voltage doubler, a voltage regulator circuit and a Nickel Metal Hydride (NiMH) battery.

In the conditioning circuitry shown in Figure 15, the AC signal from the harvester is converted into a DC signal through a passive half-wave voltage doubler. The voltage doubler converts the two-phase AC signal produced by the harvester (positive and negative voltage signals) into a DC signal and doubles its magnitude. The diodes prevent signal cancellation from the production of equal and opposite voltages when an array of harvesters is used. A half-wave voltage doubler is chosen over a full wave rectifier due to the fewer diodes required, leading to lower diode voltage drop and higher rectification efficiency. The voltage doubler is shown consisting of two diodes D_1_ and D_2_, and an output capacitor C_2_ to filter and smooth the output of the doubler.

In order to provide a suitably constant DC voltage to charge the NiMH battery, a voltage regulator is required. A LT3009 regulator (Linear Technology Inc., Milpitas, CA, USA) was chosen for this circuit due to its very low quiescent current (3 µA), the low minimum input voltage of 1.6 V, its adjustable output voltage, and the relatively low number of components required to make the integrated circuit (IC) operate. Input and output capacitors, C_3_ and C_4_, are required to filter the noise of the input and output signals to the regulator. The output voltage of the regulator can be adjusted by varying the ratio of the control resistors R_1_ and R_2_. For this study, R_1_ set to 705 kΩ and R_2_ is set to 604 kΩ to set the voltage regulator to 1.3 V.

The output of the voltage regulator must be current limited, with a limiting resistor R_3_, to prevent damage to the NiMH battery as well as the regulator (maximum 20 mA only). A NiMH battery was chosen due to the low charging voltage (1.3 V–1.4 V per cell) than other types of batteries, such as Li-ion (3.7 V per cell), Li-polymer (4.2 V per cell), and Lead acid (2.4 V per cell) [43]. Although a NiMH battery reduces the required voltage threshold to charge the battery, limiting the energy generation requirements of the harvesters, it is the least evolved of the battery technologies available. NiMH comparatively has the lowest energy density, the largest degree of self-discharge, and the lowest life cycle in comparison to Li-ion and Li-polymer batteries.

### 3.3. Feasibility Study of the Conditioning Circuitry

A feasibility study of the conditioning circuitry was undertaken to overcome the challenges encountered in the development of the designed conditioning circuitry shown in Figure 15. These included maximizing the efficiency and charging current delivered to the battery requiring the use of low activation voltage and low current consumption components. Due to excessive losses, high voltage drops, and poor efficiency, components such as full wave rectifiers and battery management circuits with embedded logic were not suitable for this low power application. The feasibility study focused on the efficiency and battery charging current of conditioning circuitry in response to different input signals’ characteristics such as, the input voltage(varied from 3 to 5 V peak-to-peak, the input frequency (varied from 100 to 300 Hz), and the NiMH battery voltage (varied from 1 to 1.25 V). The range of input frequency and voltage considered in this work complies with those reported in the literature. For example, common input vibrations from household appliances are between 60 and 200 Hz [3,44]. A comparison between different piezoelectric energy harvesters is shown in [4], and the operating voltage values are presented. Both simulation and experimental measurements were used to determine the battery charging current and overall efficiency for a variety of combinations of these variables.

**Figure 16:**
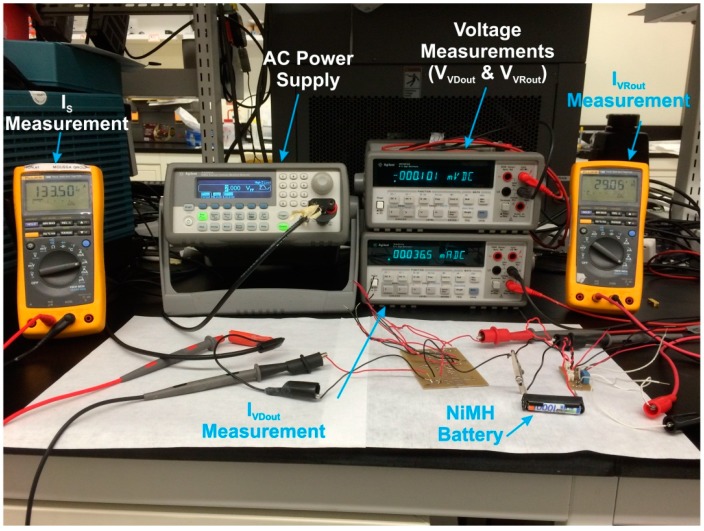
The experimental setup for the proposed electrical interface.

The simulation was performed with LTSpice IV (Linear Technology Inc. Milpitas, CA, USA) to utilize standard models of the chosen voltage regulator from Linear Technology. It is a high performance SPICE simulator allowing user to view the regulator output waveforms in a few minutes. The experimental setup is shown in Figure 16, consisting of an 33220A AC supply (Agilent, Santa Clara, CA, USA), Agilent 34401A voltage meters, 189 RMS multimeters (Fluke, Mississauga, ON, Canada), and an HR-4U 1000 mAh NiMH battery (Sanyo, Newark, NJ, USA).

**Figure 17:**
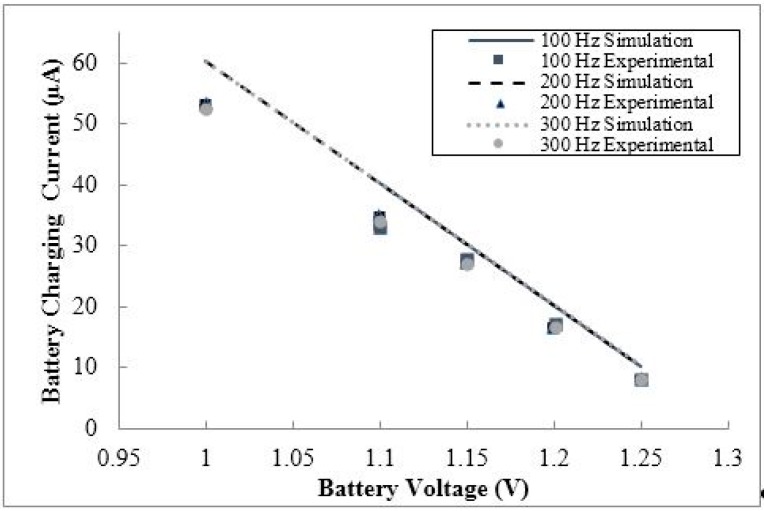
The simulated and experimental battery current behavior *versus* battery voltage for three input frequency cases (100 Hz, 200 Hz, and 300 Hz). The correlation of results for all input frequencies suggests that the battery current does not depend on input frequency.

The initial stage of the feasibility study dealt with the effect of the battery voltage on the performance of the circuitry. In general, it was observed that the voltage of the NiMH battery was the only parameter in the study that affected the current delivered to the battery from the conditioning circuitry, as shown in Figure 17. The internal resistor of the NiMH is constant (approximate 0.17 Ω) though the entire discharge stage.

As shown in Figure 17, any variation in the input frequency to the conditioning circuit does not affect the current delivered to the battery. The same conclusion was found when varying input frequency for set levels of input voltage. Therefore, it can be concluded that the current supplied to the battery is insensitive to the input signal supplied to the conditioning circuitry. This result is logical, since the voltage regulator and current limiting resistance would regulate the current and voltage supplied to the battery. As shown in Figure 17, the current delivered to the battery is maximized at low battery voltage due to Ohm’s law. It was found that the maximum the maximum battery current was 51.7 µA, at an input frequency of 100 Hz, input voltage of 4 V p-p, and battery voltage of 1 V.

**Figure 18:**
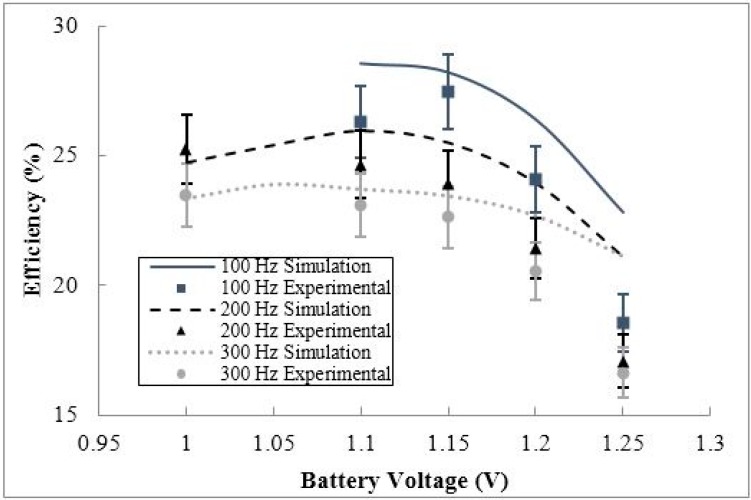
The simulated and experimental efficiency behavior *versus* battery voltage for three input frequency cases (100 Hz, 200 Hz, and 300 Hz).

To examine the effect of battery voltage on the efficiency of the conditioning circuit, the circuit efficiency was evaluated for a variety of input voltages and frequencies. The efficiency behavior of the circuit *vs.* the battery voltage for varying input frequencies is shown in Figure 18.

**Figure 19:**
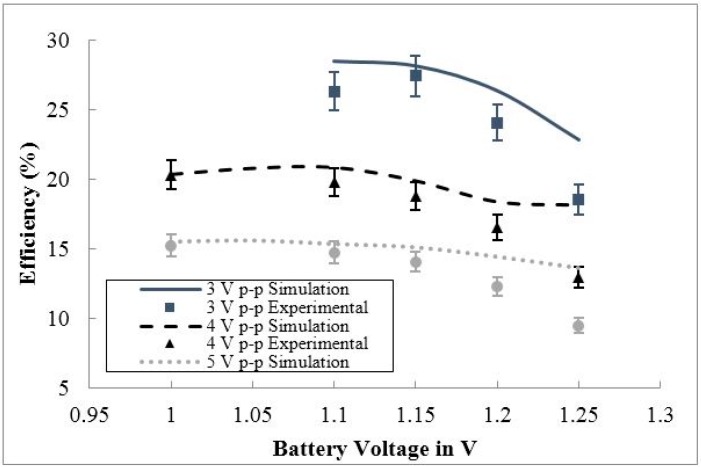
The simulated and experimental efficiency behavior *vs.* battery voltage for three input voltage cases (3 V p-p, 4 V p-p, and 5 V p-p).

As shown in Figure 18, the efficiency of the conditioning circuit was measured at 16.6% to 27.5% for the three input frequency cases. The overall circuit efficiency decreases as the input frequency increases. This occurs due to the higher DC voltage output from the voltage doubler leading to a higher voltage difference between the input and output voltages on the voltage regulator. In the ideal situation, the input power (I_in_ × V_in_) is equal to the output power (I_out_ × V_out_). Therefore, the ratio between I_in_ and the quiescent current of the voltage regulator affects the efficiency of the regulator. In the 100 Hz case, the circuit was not able to operate at battery voltages lower than 1.1 V due to the output DC voltage level from the voltage doubler dropping below the minimum 1.6 V required by the voltage regulator. To aid in the operation of the circuit at low input frequencies, the input voltage or the current limiting resistance should increase. This will not only allow for the operation of the circuit at low input voltage frequency, but will lead to a higher voltage doubler efficiency. The voltage regulator dominates the overall efficiency of the circuit, varying between 35% and 72% efficiency, while the voltage doubler varies between 41% and 52% efficiency. Additionally, the overall circuit efficiency can be improved by increasing the efficiency of voltage doubler and voltage regulator circuits. The discrepancy between the simulation and experimental results are expected, and it is mainly due to the tolerance of the different components used in the experiment, also due to un-modeled physical parameters as the NiMH battery internal resistance. In addition, experimental errors shown in Figure 18, and following figures, are calculated based on the measurement errors in each quantity required to capture the efficiency. Lastly, in general, the efficiency of the circuit decreases as the battery voltage increases for all frequency cases examined, due to the reduction of the battery charging current as seen in Figure 17. The efficiency behavior of the circuit *vs.* the battery voltage for varying input voltages is shown in Figure 19.

Figure 19 shows the overall efficiency of the proposed circuit for varying battery voltages for three cases of input voltage to the circuit. The overall efficiency for the proposed circuit was measured between 9.4% and 27% for the examined input voltage cases. The efficiency of the circuit was consistently higher for lower applied input voltages. As with the case in efficiency study in Figure 18, this input voltage-dependent behavior occurs due to higher DC voltage output from the voltage doubler, leading to a higher quiescent current and lower regulator efficiency. Additionally, the voltage regulator dominates the overall efficiency of the circuit, varying between 20% and 73% efficiency, while the voltage doubler varies between 42% and 50% efficiency. As with the frequency-based study, the low voltage case of 3 V peak-to-peak could not activate the conditioning circuit under a battery voltage of 1.1 V due an insufficient voltage supplied to the voltage regulator. An increase in input frequency would aid in the low voltage operation by increasing the voltage doubler output voltage. The discrepancy between the simulation and experimental results are due to the tolerance of the components used in the experiment and un-modeled NiMH battery internal resistance.

**Figure 20:**
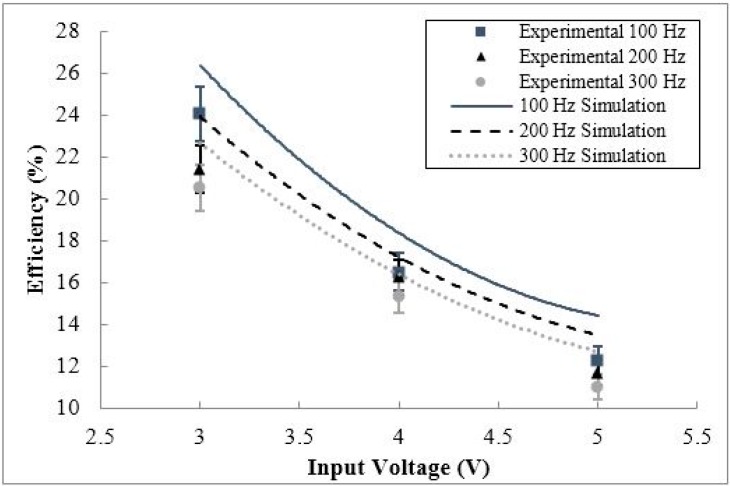
The simulated and experimental efficiency of the conditioning circuit for varying input voltage, for three input frequency cases (100 Hz, 200 Hz, and 300 Hz).

**Figure 21:**
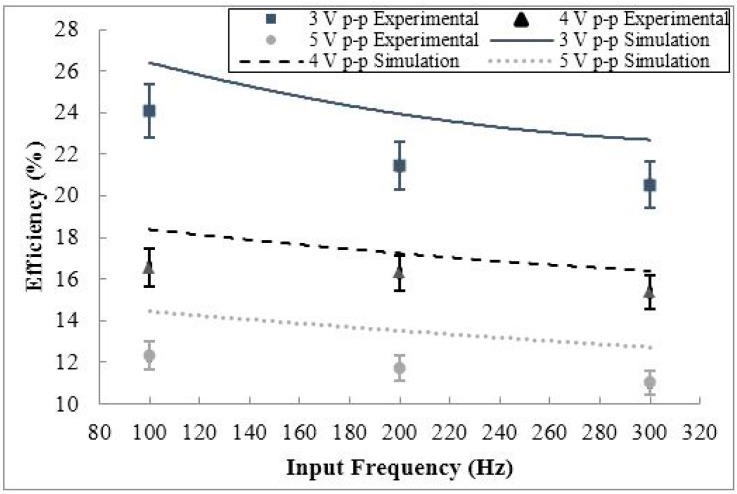
The simulated and experimental efficiency of the conditioning circuit for varying input frequency, for three input voltage cases (3 V p-p, 4 V p-p, and 5 V p-p).

From the consistency of the efficiency behavior of the circuit for a variety of input frequency and input voltage cases in Figure 18 and Figure 19 for varying battery voltage, it can be concluded that at a specific battery voltage, lower input frequency and voltage will lead to a higher circuit efficiency. To understand the interdependency of input frequency and voltage on the efficiency of the conditioning circuit, the efficiency of the circuit will be examined for varying input voltages and frequencies at a fixed battery voltage. The efficiency behavior for varying input voltage and frequency, at a fixed nominal battery voltage of 1.2 V can be seen in Figure 20 and Figure 21, respectively.

As shown in Figure 20, at a constant input frequency, the circuit efficiency increases with decreasing input voltage due to the higher voltage regulator efficiency for lower DC voltages. Additionally, at a constant input voltage, the circuit efficiency increases with decreasing input frequency due to higher voltage ripples associated with lower frequency operating conditions lowering the DC voltage inputted into the voltage regulator, increasing regulator efficiency. As shown in Figure 21, the overall efficiency decreases as the input voltage and frequency increases due to the increased quiescent current absorbed by the voltage regulator. The maximum circuit efficiency for this study was 24.1% at an input voltage of 3 V p-p, an input frequency of 100 Hz, at the constant battery voltage of 1.2 V. Taking the two cases outlined in Figure 20 and Figure 21, it is clear that the highest efficiency occurs at the lowest input voltage and frequency for constant battery voltages. Taking the dependence of circuit efficiency on the battery voltage into account, the highest possible efficiency would occur at the lowest operational battery voltage, input voltage, and input frequency.

## 4. Conclusions

The methodologies used and challenges encountered in the microfabrication and integration of fixed-fixed folded spring energy harvesters designed and characterized in [5] have been discussed in detail. A variety of challenges were overcome in the fabrication of the energy harvesters, such as the diagnosis and rectification of sol-gel PZT film quality and adhesion issues, including the use of more robust adhesion materials. Additionally, the use of a carrier wafer allowed for the use of a carrier wafer to promote non-invasive cleaving of the harvesters. A complete packaging and integration methodology was developed to facilitate testing the harvesters under a base vibration. This included developing a packaging methodology using printed circuit boards and wirebonding to allow for the electrical characterization of the harvester without relying on a traditional probe station.

Two conditioning circuits were developed to electrically characterize the harvester and to store the harvested energy in a NiMH battery. The initial simple conditioning circuit was useful to measure the total available power from the harvester. The second conditioning circuit developed allowed for the creation of a complete energy harvesting system, consisting of the harvester, a voltage doubler, a voltage regulator and a NiMH battery. The biggest challenge in the development of the conditioning circuitry is maximizing the efficiency and current stored in the battery. The maximum circuit efficiency is 27.5%, at an input frequency of 100 Hz, input voltage of 3 V p-p, battery voltage of 1.15 V, and current limiting resistance of 5 kΩ. The battery charging current for this case is 27.9 µA, with voltage doubler efficiency of 47.5%, and voltage regulator efficiency of 67.9%. However, the maximum battery charging current was found to be 51.7 µA at an input frequency of 100 Hz, input voltage of 4 V p-p, battery voltage of 1 V, and current limiting resistance of 5 kΩ. The overall efficiency for this case is 20.32%. The voltage doubler efficiency is 41.9% and the voltage regulator efficiency is 62.9%. It can be concluded that the maximum efficiency does not correlate to the maximum charging current supplied to the battery. Conversely, the maximum charging current can be achieved at the expense of the overall efficiency. The charging current supplied to the battery is only effected by the battery voltage, increasing with decreasing battery voltage. The efficiency of the designed conditioning circuit is increased with decreasing input voltage, input frequency, and battery voltage. The efficiency and charging current must be balanced to achieve a high output and a reasonable output current. Transitioning to active conditioning circuitry in future iterations of the energy harvestings system should increase efficiency and current supplied to the battery. The development of the complete energy harvesting system allows for the direct integration of the energy harvesting technology previously developed into existing power management schemes for wireless sensing.
